# Crystal structure of dicesium hydrogen citrate from laboratory single-crystal and powder X-ray diffraction data and DFT comparison

**DOI:** 10.1107/S2056989017000792

**Published:** 2017-01-20

**Authors:** Alagappa Rammohan, Amy A. Sarjeant, James A. Kaduk

**Affiliations:** a Atlantic International University, Honolulu HI , USA; b Department of Chemistry, Northwestern University, Evanston IL , USA; c Illinois Institute of Technology, Department of Chemistry, 3101 S. Dearborn St., Chicago IL 60616, USA

**Keywords:** crystal structure, powder diffraction, density functional theory, citrate, cesium

## Abstract

The crystal structure of dicesium hydrogen citrate, 2Cs^+^·C_6_H_6_O_7_^2−^, has been solved using laboratory X-ray single-crystal diffraction data, refined using laboratory powder X-ray data, and optimized using density functional techniques. The Cs^+^ cation is nine-coordinate, with a bond-valence sum of 0.92 valence units. The CsO_9_ coordination polyhedra share edges and corners to form a three-dimensional framework. The citrate anion is located on a mirror plane. Its central hy­droxy/carboxyl­ate O—H⋯O hydrogen bond is short, and (unusually) inter­molecular. The centrosymmetric end-end carboxyl­ate hydrogen bond is exceptionally short (O⋯O = 2.416 Å) and strong. These hydrogen bonds contribute 16.5 and 21.7 kcal mol^−1^, respectively, to the crystal energy. The hydro­phobic methyl­ene groups occupy pockets in the framework.

## Abstract 2

The crystal structure of dicesium hydrogen citrate has been solved using laboratory X-ray single-crystal diffraction data, refined using laboratory powder data, and optimized using density functional techniques.

## Chemical context   

In the course of a systematic study of the crystal structures of group 1 (alkali metal) citrate salts to understand the anion’s conformational flexibility, ionization, coordination tendencies, and hydrogen bonding, we have determined several new crystal structures. Most of the new structures were solved using X-ray powder diffraction data (laboratory and/or synchrotron), but single crystals were used where available. The general trends and conclusions about the sixteen new compounds and twelve previously determined structures are being reported separately (Rammohan & Kaduk, 2017*a* ▸). Eleven of the new structures – NaKHC_6_H_5_O_7_, NaK_2_C_6_H_5_O_7_, Na_3_C_6_H_5_O_7_, NaH_2_C_6_H_5_O_7_, Na_2_HC_6_H_5_O_7_, K_3_C_6_H_5_O_7_, Rb_2_HC_6_H_5_O_7_, Rb_3_C_6_H_5_O_7_(H_2_O), Rb_3_C_6_H_5_O_7_, Na_5_H(C_6_H_5_O_7_)_2_, and CsH_2_C_6_H_5_O_7_ – have been published recently (Rammohan & Kaduk, 2016*a* ▸,*b* ▸,*c* ▸,*d* ▸,*e* ▸, 2017*b* ▸,*c* ▸,*d* ▸,*e* ▸,*f* ▸, Rammohan *et al.*, 2016 ▸), and two additional structures – KH_2_C_6_H_5_O_7_ and KH_2_C_6_H_5_O_7_(H_2_O)_2_ – have been communicated to the Cambridge Structural Database (CSD) (Kaduk & Stern, 2016*a* ▸,*b* ▸). We report here synthesis and crystal structure of another alkali metal citrate salt, 2Cs^+^·HC_6_H_5_O_7_^2−^.

## Structural commentary   

**Figure 1:**
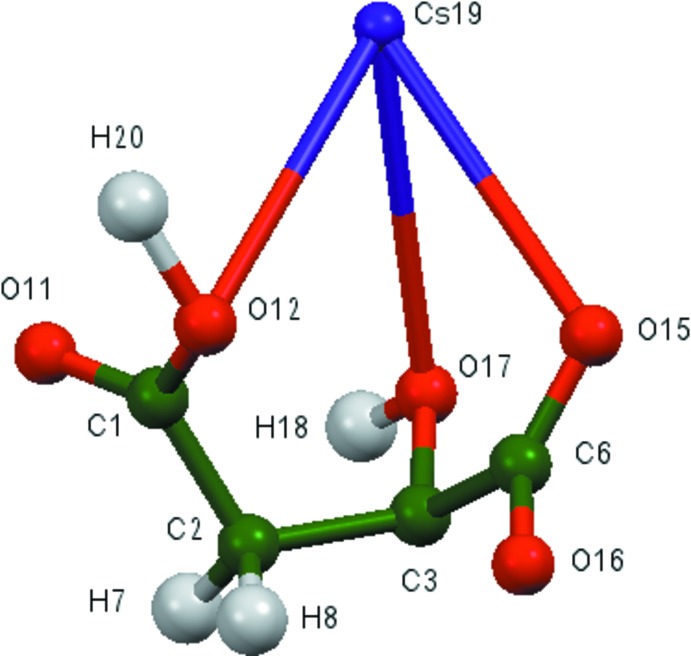
The asymmetric unit of the title compound, with the atom numbering. The atoms are represented by 50% probability spheroids.

**Figure 2:**
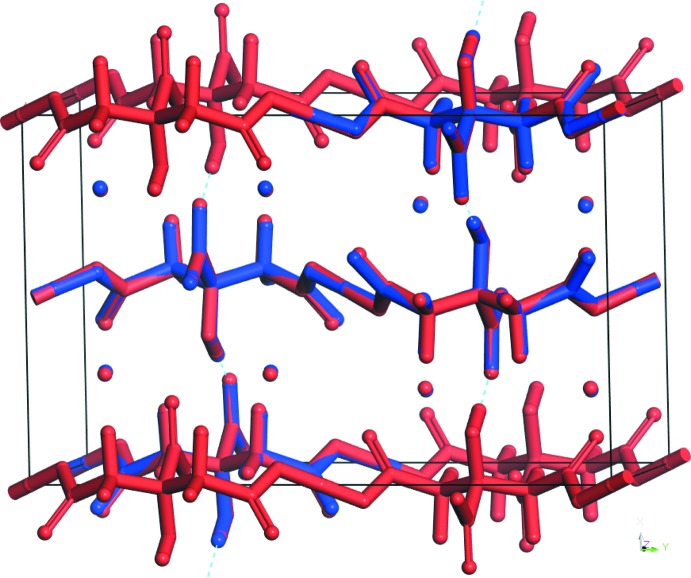
Comparison of the refined and optimized structures of dicesium hydrogen citrate. The refined structure is in red, and the DFT-optimized structure is in blue.

The asymmetric unit of the title compound is shown in Fig. 1 ▸. The root-mean-square deviation of the non-hydrogen atoms in the experimentally determined and in the DFT-optimized structures is 0.098 Å (Fig. 2 ▸). The largest differences are 0.13 Å, at Cs19 and O11. This good agreement provides strong evidence that the experimentally determined structure is correct (van de Streek & Neumann, 2014 ▸). The following discussion uses the DFT-optimized structure.
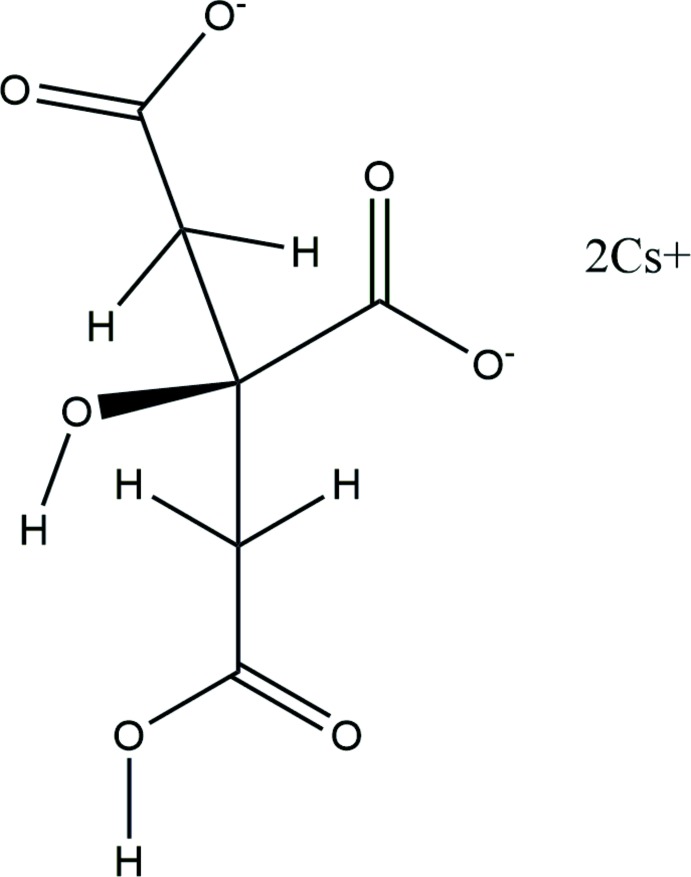


Most of the bond lengths, bond angles, and torsion angles fall within the normal ranges indicated by a *Mercury* Mogul geometry check (Macrae *et al.*, 2008 ▸). The C1—C2—C3 angle of 114.1° is flagged as unusual (average = 104.0 (32), *Z*-score = 3.1). The Cs^+^ cation is 9-coordinate, with a bond-valence sum of 0.92 valence units. The location of the citrate anion on a mirror plane and the coordination of all seven oxygen atoms to Cs^+^ cations presumably are the source of the slight distortion. The citrate anion occurs in the *trans,trans* conformation, which is one of the two low-energy conformations of an isolated citrate moiety. The citrate anion triply chelates to two Cs^+^ cations through O12, O17, and O15. The citrate also chelates through O12/O16, O15/O17, and O15/O16. The Mulliken overlap populations and atomic charges indicate that the metal-oxygen bonding is ionic. The Bravais–Friedel–Donnay–Harker (Bravais, 1866 ▸; Friedel, 1907 ▸; Donnay & Harker, 1937 ▸) morphology is blocky, with {020} as major faces. A 4th-order spherical harmonic model was included in the refinement. The texture index was 1.016, indicating that preferred orientation was slight in the rotated flat-plate specimen.

## Supra­molecular features   

**Figure 3:**
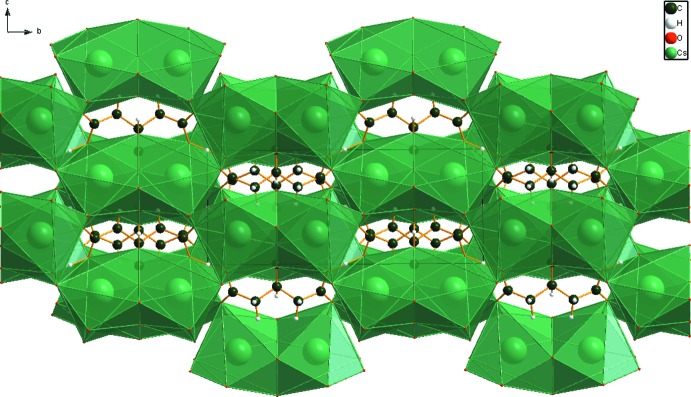
Crystal structure of Cs_2_HC_6_H_5_O_7_, viewed down the *a*-axis. CsO_9_ polyhedra are green.

The CsO_9_ coordination polyhedra share edges and corners to form a three-dimensional framework (Fig. 3 ▸). The central hy­droxy/carboxyl­ate O—H⋯O hydrogen O17—H18⋯O16 is short, and (unusually) inter­molecular. The centrosymmetric end-end O12—H20—O12 hydrogen bond (with H20 situated on an inversion center) is exceptionally short and strong (Table 1 ▸). By the correlation of Rammohan & Kaduk (2017*a* ▸), these hydrogen bonds contribute 16.5 and 21.7 kcal mol^−1^ to the crystal energy. The hydro­phobic methyl­ene groups occupy pockets in the framework (Fig. 3 ▸).

## Database survey   

Details of the comprehensive literature search for citrate structures are presented in Rammohan & Kaduk (2017*a* ▸). A reduced cell search of the cell of dicesium hydrogen citrate in the Cambridge Structural Database (Groom *et al.*, 2016 ▸) (increasing the default tolerance from 1.5 to 2.0%) yielded 100 hits, but combining the cell search with the elements C, H, Cs, and O only yielded no hits.

## Synthesis and crystallization   

Citric acid monohydrate, H_3_C_6_H_5_O_7_(H_2_O), (2.0796 g, 10.0 mmol) was dissolved in 20 ml deionized water. Cs_2_CO_3_ (3.2582 g, 10.0 mmol, Sigma–Aldrich) was added to the citric acid solution slowly with stirring. The resulting clear colourless solution was evaporated to dryness in a 333 K oven. Single crystals were isolated from the colourless solid.

## Refinement   

A single crystal was mounted in inert oil and transferred to the cold gas stream of a Bruker Kappa APEX CCD area detector system equipped with a Cu K*α* sealed tube with MX optics. Despite suggestions from multiple programs that the space group was *Pnma*, all attempts to refine the structure in this space group yielded unreasonable disorder and non-positive-definite displacement coefficients. Presumably the poor crystal quality and/or twinning were the source of the problems. The best refinement using single crystal data was obtained using space group *P*2_1_2_1_2_1_.

A portion of the sample was ground in a mortar and pestle, and blended with NIST SRM 640b silicon inter­nal standard. The powder pattern indicated that the sample contained about 24 wt% CsHC_6_H_5_O_7_ (Rammohan & Kaduk, 2017*f* ▸), which was included as phase 2 in the refinement. The Si inter­nal standard was included as phase 3.

**Figure 4:**
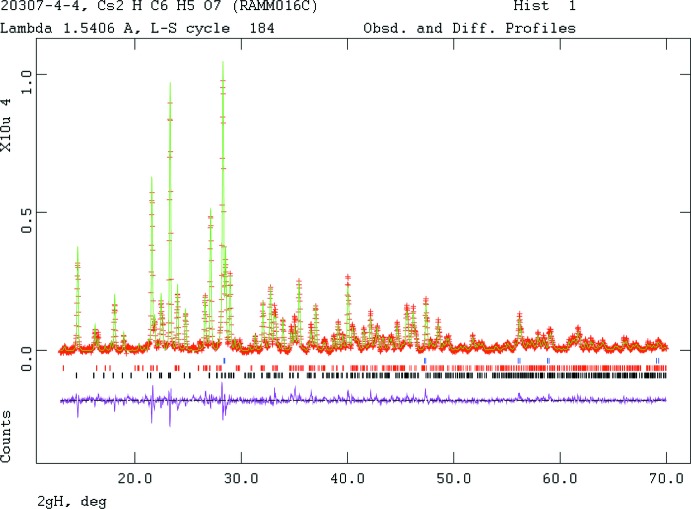
Rietveld plot for the refinement of Cs_2_HC_6_H_5_O_7_. The red crosses represent the observed data points, and the green line is the calculated pattern. The magenta curve is the difference pattern, plotted at the same scale as the other patterns. The row of black tick marks indicates the reflection positions, the row of red tick marks indicates the positions of the CsH_2_C_6_H_5_O_7_ impurity peaks, and the blue tick marks indicate the Si inter­nal standard peaks.

Initial Rietveld refinements used the single crystal *P*2_1_2_1_2_1_ model, but were unstable. The ADDSYM module of *PLATON* (Spek, 2009 ▸) suggested the presence of an additional center of symmetry, and that the correct space group was *Pnma* (with a transformation of axes). Refinement in the higher-symmetry space group was uneventful. Pseudo-Voigt profile coefficients were as parameterized in Thompson *et al.* (1987 ▸) with profile coefficients for Simpson’s rule integration of the pseudo-Voigt function according to Howard (1982 ▸). The asymmetry correction of Finger *et al.* (1994 ▸) was applied, and microstrain broadening by Stephens (1999 ▸). The structure was refined by the Rietveld method using *GSAS/EXPGUI* (Larson & Von Dreele, 2004 ▸; Toby, 2001 ▸). All C—C and C—O bond lengths were restrained, as were all bond angles. The hydrogen atoms were included at fixed positions, which were recalculated during the course of the refinement using *Materials Studio* (Dassault Systemes, 2014 ▸). The limited resolution of the powder data precluded refining displacement coefficients, which were fixed at typical values for alkali metal citrates. Diffraction data are displayed in Fig. 4 ▸. Crystal data, data collection and structure refinement details are summarized in Table 2 ▸.

## DFT calculations   

After the Rietveld refinement, a density functional geometry optimization (fixed experimental unit cell) was carried out using CRYSTAL14 (Dovesi *et al.*, 2014 ▸). The basis sets for the C, H, and O atoms were those of Peintinger *et al.* (2012 ▸), and the basis set for Cs was that of Sophia *et al.* (2014 ▸). The calculation was run on eight 2.1 GHz Xeon cores (each with 6 Gb RAM) of a 304-core Dell Linux cluster at IIT, used 8 *k*-points and the B3LYP functional, and took about 13 h. The *U*_iso_ values from the Rietveld refinement were assigned to the optimized fractional coordinates.

## Supplementary Material

Crystal structure: contains datablock(s) RAMM016C_publ, ramm016c_DFT, RAMM016C_overall, RAMM016C_phase_1, RAMM016C_phase_2, RAMM016C_phase_3, RAMM016C_p_01. DOI: 10.1107/S2056989017000792/wm5358sup1.cif

Supporting information file. DOI: 10.1107/S2056989017000792/wm5358RAMM016C_phase_3sup2.cml

CCDC references: 1527789, 1527790, 1527791, 1527792

Additional supporting information:  crystallographic information; 3D view; checkCIF report

## Figures and Tables

**Table 1:** Hydrogen-bond geometry (Å, °)

*D*—H⋯*A*	*D*—H	H⋯*A*	*D*⋯*A*	*D*—H⋯*A*
O12—H20⋯O12^i^	1.208	1.208	2.416	180.0
O17—H18⋯O16^ii^	0.999	1.634	2.632	178.2

Symmetry codes: (i) 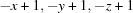; (ii) 
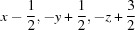
.

**Table 2:** Experimental details

	Phase 1	Phase 2
Crystal data		
Chemical formula	2Cs^+^·C_6_H_6_O_7_^2−^	C_6_H_7_CsO_7_
*M*_r_	455.92	324.02
Crystal system, space group	Orthorhombic, *P* *n* *m* *a*	Orthorhombic, *P* *n* *a*2_1_
Temperature (K)	300	300
*a*, *b*, *c* (Å)	9.8466 (3), 15.8872 (5), 6.5959 (2)	8.7362, 20.5351, 5.1682
*V* (Å^3^)	1031.82 (6)	927.17
*Z*	4	4
Radiation type	*K*α_1_, *K*α_2_, λ = 1.540629, 1.544451 Å	*K*α_1_, *K*α_2_, λ = 1.540629, 1.544451 Å
Specimen shape, size (mm)	Flat sheet, 24 × 24	Flat sheet, 24 × 24

Data collection		
Diffractometer	Bruker D2 Phaser	Bruker D2 Phaser
Specimen mounting	Standard PMMA holder	Standard PMMA holder
Data collection mode	Reflection	Reflection
Scan method	Step	Step
2θ values (°)	2θ_min_ = 5.042 2θ_max_ = 70.050 2θ_step_ = 0.020	2θ_min_ = 5.042 2θ_max_ = 70.050 2θ_step_ = 0.020

Refinement		
*R* factors and goodness of fit	*R*_p_ = 0.050, *R*_wp_ = 0.062, *R*_exp_ = 0.030, *R*(*F* ^2^) = 0.081, χ^2^ = 4.494	*R*_p_ = 0.050, *R*_wp_ = 0.062, *R*_exp_ = 0.030, *R*(*F* ^2^) = 0.081, χ^2^ = 4.494
No. of parameters	57	57
No. of restraints	18	18

The same symmetry and lattice parameters were used for the DFT calculation. Computer programs: *DIFFRAC.Measurement* (Bruker, 2009 ▸), *SHELXT* (Sheldrick, 2015 ▸), *GSAS* (Larson & Von Dreele, 2004 ▸), *DIAMOND* (Crystal Impact, 2015 ▸), *publCIF* (Westrip, 2010 ▸).

## supplementary crystallographic information

### Crystal data

**Table:**

C_6_H_6_Cs_2_O_7_	*b* = 15.8872 Å
*M**_r_* = 455.92	*c* = 6.5959 Å
Orthorhombic, *P**n**m**a*	*V* = 1031.82 Å^3^
Hall symbol: -P 2ac 2n	*Z* = 4
*a* = 9.8466 Å	*T* = 300 K

### Data collection

**Table:**

*h* = →	*l* = →
*k* = →	

### Fractional atomic coordinates and isotropic or equivalent isotropic displacement parameters (Å^2^)

**Table:**

	*x*	*y*	*z*	*U*_iso_*/*U*_eq_	
C1	0.48590	0.40995	0.73839	0.03000*	
C2	0.53924	0.32807	0.82202	0.03000*	
H7	0.49922	0.81932	0.02513	0.03900*	
H8	0.35061	0.83203	0.17117	0.03900*	
O11	0.39754	0.45079	0.82841	0.03000*	
O12	0.54129	0.43424	0.56822	0.03000*	
Cs19	0.28894	0.39193	0.26540	0.02000*	
C3	0.50141	0.25000	0.69412	0.03000*	
C6	0.58468	0.25000	0.49542	0.03000*	
O15	0.52651	0.25000	0.32675	0.03000*	
O16	0.71279	0.25000	0.52034	0.03000*	
O17	0.36003	0.25000	0.64658	0.03000*	
H18	0.30582	0.25000	0.77454	0.03900*	
H20	0.50000	0.50000	0.50000	0.03900*	

### Bond lengths (Å)

**Table:**

C1—C2	1.507	O12—H20	1.208
C1—O11	1.237	C3—C2^iii^	1.546
C1—O12	1.306	C3—C6	1.546
C2—C3	1.546	C3—O17	1.427
C2—H7^i^	1.086	C6—O15	1.251
C2—H8^i^	1.087	C6—O16	1.272
H7—C2^ii^	1.086	O17—H18	0.999
H8—C2^ii^	1.087	H20—O12^iv^	1.208

Symmetry codes: (i) −*x*+1, *y*−1/2, −*z*+1; (ii) −*x*+1, *y*+1/2, −*z*+1; (iii) *x*, −*y*+1/2, *z*; (iv) −*x*+1, −*y*+1, −*z*+1.

### Hydrogen-bond geometry (Å, º)

**Table:**

*D*—H···*A*	*D*—H	H···*A*	*D*···*A*	*D*—H···*A*
O12—H20···O12^iv^	1.208	1.208	2.416	180.0
O17—H18···O16^v^	0.999	1.634	2.632	178.2

Symmetry codes: (iv) −*x*+1, −*y*+1, −*z*+1; (v) *x*−1/2, −*y*+1/2, −*z*+3/2.

## References

[bb1] Bravais, A. (1866). In *Etudes Cristallographiques*. Paris: Gauthier Villars.

[bb2] Bruker (2009). *DIFFRAC. Measurement*. Bruker AXS Inc., Madison Wisconsin, USA.

[bb3] Crystal Impact (2015). *DIAMOND*. Crystal Impact GbR, Bonn, Germany.

[bb4] Dassault Systemes (2014). *Materials Studio*. BIOVIA, San Diego, California, USA.

[bb5] Donnay, J. D. H. & Harker, D. (1937). *Am. Mineral.* **22**, 446–467.

[bb6] Dovesi, R., Orlando, R., Erba, A., Zicovich-Wilson, C. M., Civalleri, B., Casassa, S., Maschio, L., Ferrabone, M., De La Pierre, M., D’Arco, P., Noël, Y., Causà, M., Rérat, M. & Kirtman, B. (2014). *Int. J. Quantum Chem.* **114**, 1287–1317.

[bb7] Finger, L. W., Cox, D. E. & Jephcoat, A. P. (1994). *J. Appl. Cryst.* **27**, 892–900.

[bb8] Friedel, G. (1907). *Bull. Soc. Fr. Mineral.* **30**, 326–455.

[bb9] Groom, C. R., Bruno, I. J., Lightfoot, M. P. & Ward, S. C. (2016). *Acta Cryst.* B**72**, 171–179. 10.1107/S2052520616003954 PMC4822653 27048719

[bb10] Howard, C. J. (1982). *J. Appl. Cryst.* **15**, 615–620.

[bb11] Kaduk, J. A. & Stern, C. (2016*a*). CSD Communication 1446457–1446458.

[bb12] Kaduk, J. A. & Stern, C. (2016*b*). CSD Communication 1446460–1446461.

[bb13] Larson, A. C. & Von Dreele, R. B. (2004). *General Structure Analysis System*, (*GSAS*). Report LAUR, 86–784 Los Alamos National Laboratory, New Mexico, USA.

[bb14] Macrae, C. F., Bruno, I. J., Chisholm, J. A., Edgington, P. R., McCabe, P., Pidcock, E., Rodriguez-Monge, L., Taylor, R., van de Streek, J. & Wood, P. A. (2008). *J. Appl. Cryst.* **41**, 466–470.

[bb15] Peintinger, M. F., Vilela Oliveira, D. & Bredow, T. (2012)*. Comput. Chem.*, doi: 10.1002/jcc.23153.

[bb16] Rammohan, A. & Kaduk, J. A. (2016*a*). *Acta Cryst.* E**72**, 170–173. 10.1107/S2056989016000232 PMC4770952 26958380

[bb17] Rammohan, A. & Kaduk, J. A. (2016*b*). *Acta Cryst.* E**72**, 403–406. 10.1107/S2056989016002966 PMC4778822 27006817

[bb18] Rammohan, A. & Kaduk, J. A. (2016*c*). *Acta Cryst.* E**72**, 793–796. 10.1107/S2056989016007453 PMC4908562 27308044

[bb19] Rammohan, A. & Kaduk, J. A. (2016*d*). *Acta Cryst.* E**72**, 854–857. 10.1107/S2056989016008343 PMC4908552 27308058

[bb20] Rammohan, A. & Kaduk, J. A. (2016*e*). *Acta Cryst.* E**72**, 1159–1162. 10.1107/S2056989016011506 PMC4971862 27536403

[bb21] Rammohan, A. & Kaduk, J. A. (2017*a*). *Acta Cryst. B.* Submitted.

[bb22] Rammohan, A. & Kaduk, J. A. (2017*b*). *Acta Cryst.* E**73**, 92–95. 10.1107/S2056989016020168 PMC5209781 28083145

[bb23] Rammohan, A. & Kaduk, J. A. (2017*c*). *Acta Cryst.* E**73**, 227–230. 10.1107/S2056989017000743 PMC5290571 28217348

[bb24] Rammohan, A. & Kaduk, J. A. (2017*d*). *Acta Cryst.* E**73**, 250–253. 10.1107/S2056989017001086 PMC5290576 28217353

[bb25] Rammohan, A. & Kaduk, J. A. (2017*e*). *Acta Cryst.* E**73**, 286–290. 10.1107/S2056989017001256 PMC5290583 28217360

[bb26] Rammohan, A. & Kaduk, J. A. (2017*f*). *Acta Cryst.* E**73**, 133–136. 10.1107/S2056989017000135 PMC5290550 28217327

[bb27] Rammohan, A., Sarjeant, A. A. & Kaduk, J. A. (2016). *Acta Cryst.* E**72**, 943–946. 10.1107/S2056989016009014 PMC4992911 27555936

[bb28] Sheldrick, G. M. (2015). *Acta Cryst.* A**71**, 3–8.

[bb29] Sophia, G., Baranek, P., Sarrazin, M., Rerat, M. & Dovesi, R. (2014). *Systematic influence of atomic substitution on the phase diagram of ABO_3_ ferroelectric perovskites.*

[bb30] Spek, A. L. (2009). *Acta Cryst.* D**65**, 148–155. 10.1107/S090744490804362X PMC2631630 19171970

[bb31] Stephens, P. W. (1999). *J. Appl. Cryst.* **32**, 281–289.

[bb32] Streek, J. van de & Neumann, M. A. (2014). *Acta Cryst.* B**70**, 1020–1032. 10.1107/S2052520614022902 PMC4468513 25449625

[bb33] Thompson, P., Cox, D. E. & Hastings, J. B. (1987). *J. Appl. Cryst.* **20**, 79–83.

[bb34] Toby, B. H. (2001). *J. Appl. Cryst.* **34**, 210–213.

[bb35] Westrip, S. P. (2010). *J. Appl. Cryst.* **43**, 920–925.

